# Fingerprinting the Hidden Facets of Plasmonic Nanocavities

**DOI:** 10.1021/acsphotonics.2c00116

**Published:** 2022-07-27

**Authors:** Eoin Elliott, Kalun Bedingfield, Junyang Huang, Shu Hu, Bart de Nijs, Angela Demetriadou, Jeremy J Baumberg

**Affiliations:** † NanoPhotonics Centre, Cavendish Laboratory, University of Cambridge, Cambridge CB3 0HE, United Kingdom; ‡ School of Physics and Astronomy, University of Birmingham, Edgbaston, Birmingham, B15 2TT, United Kingdom

**Keywords:** plasmons, nanoparticle-on-mirror, patch antenna, quasi-normal modes, facet

## Abstract

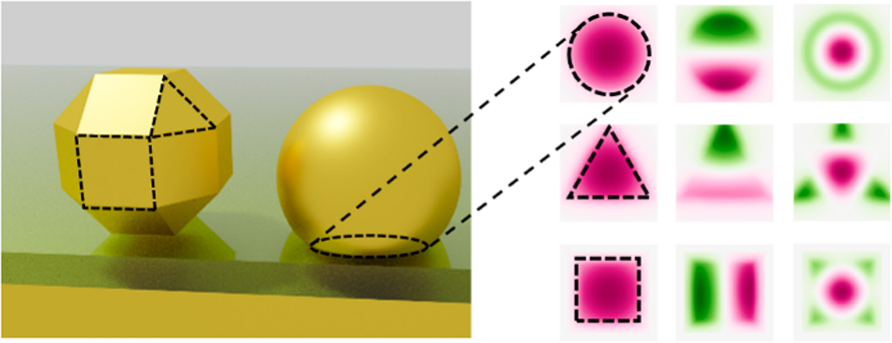

The optical properties of nanogap plasmonic cavities formed by a NanoParticle-on-Mirror (NPoM, or patch antenna) are determined here, across a wide range of geometric parameters including the nanoparticle diameter, gap refractive index, gap thickness, facet size and shape. Full understanding of the confined optical modes allows these nanocavities to be utilized in a wide range of experiments across many fields. We show that the gap thickness *t* and refractive index *n* are spectroscopically indistinguishable, accounted for by a single gap parameter *G* = *n*/*t*^0.47^. Simple tuning of mode resonant frequencies and strength is found for each quasi-normal mode, revealing a spectroscopic “fingerprint” for each facet shape, on both truncated spherical and rhombicuboctahedral nanoparticles. This is applied to determine the most likely nanoscale morphology of facets hidden below each NPoM in experiment, as well as to optimize the constructs for different applications. Simple scaling relations are demonstrated, and an online tool for general use is provided.

## Introduction

Confining light to the surface of plasmonic metals greatly increases light–matter interactions.^1^ This is further enhanced by plasmonic resonators that trap light in three dimensions, fueling advances in chemical^2^ and biological^3^ sensing, nonlinear optics,^4^ and catalysis.^5^ In contrast to microcavities that offer resonances with large quality factors *Q*, but relatively large mode volumes *V*, plasmonic nanocavities leverage the evanescent nature of localized plasmons to squeeze light into deeply subwavelength volumes. As a consequence, however, much of the optical energy is lost through inelastic scattering with the metal electrons, leading to low *Q* and small effective volume resonators. Because of this extreme localization and enhanced light-matter interaction, the spectral tuning and loss of trapped plasmonic modes is a subtle function of geometry at the nanometre scale. Understanding this interplay between nanoresonator geometry and light is vital in accounting for many widely used applications of plasmonic nanocavities such as enhancing exciton photoluminescence,^6^ nonlinear vibrational pumping,^7^ sensing, mid-infrared upconversion detectors,^8^ or hot-electron emission^9^ among many others.

To understand the details of light fields in such plasmonic nanocavities, most previous works have relied on more cumbersome simulations of highly idealized geometries and, thus, cannot easily account for the broad inhomogeneous distribution of scattering spectra observed from each nominally identical construct.^10−12^ This prevents the development of deep understanding for many light–matter effects observed experimentally. For example, simple analytical predictions are even lacking as to how modes tune when the refractive index in the subwavelength volume changes, and how this might vary for differently shaped nanoparticles (NPs).

In this work, we examine several nanocavities formed by a truncated spherical NP and a rhombicuboctahedron-shaped NP to form a wide range of nanocavity shapes. These nanocavities support tightly trapped light and allow the role of lateral confinement upon their plasmonic modes to be explored. Light can be efficiently and robustly trapped as plasmons in nanogaps based on metal–insulator–metal (MIM) configurations, and here we take a scalable widely used scheme based on the Nanoparticle-on-Mirror (NPoM) configuration.^13−18^ Nanoparticles are inevitably faceted^19^ and thus form NPoMs with a MIM plasmonic nanogap of specific lateral shape. The influence of this nanocavity shape, as well as its material properties, are the focus of this work. We investigate structures consisting of a 10–200 nm diameter metal nanoparticle (NP) spaced 0.5–10 nm away from a metal mirror separated by a dielectric spacer, which is often of molecules or inorganic layers, but also polymers, perovskites, oxides, or other materials.^20^ Although we focus here on NPoMs with both metal layers being Au (as this is the most robust commonly used plasmonic material), all our findings develop analogously in other plasmonic metals such as Ag, Al, and TiN. We also do not take into account the atomic facet plane of the Au,^21,22^ because it leads to systematic spectral shifts of only a few %, similar to other uncertainties (such as edge rounding, see below).

Usually, numerical analysis of plasmonic nanoresonators uses Finite Difference Time Domain or Finite Element Method simulations, solving Maxwell’s equations with a specific incident *E* field or emitter location, chosen to replicate an experimental system under study.^23^ This however often obscures the underlying physics of the system and brings little physical intuition, necessitating a simulation for each experiment. To compensate, several semi-analytical models have emerged in recent years which qualitatively account for the dependence on parameters including NP diameter, facet size, gap refractive index and thickness.^24−26^ These however suffer from limitations, such as multiple free fitting factors which are tuned to match the results of experiments carried out over limited parameter spaces. Additionally, they often only attempt to model the lowest energy NPoM resonance, and poorly account for higher-order modes. This is insufficient as higher-order modes are often involved in excitation or emission (as in the case of photoluminescence). These “dark” modes (which have an inherently higher *Q*) weakly scatter and play a large role in light–matter strong coupling^27^ and lasing,^28^ despite being typically disregarded. As the size, material, and shape (down to the nm-scale) significantly influence spectral tuning, isolating their effects in experiments can prove difficult.^29^

Recently, the community has begun to leverage Quasi-Normal Mode (QNM) decomposition of the electromagnetic response of plasmonic resonators,^30^ including NPoMs,^31^ to better understand their optical behavior. These QNMs are eigensolutions of Maxwell equations, producing a set of modes that are orthogonal, with parameters spanning eigenfrequencies ω̃, quality factors *Q̃*, and mode volumes *Ṽ*. These parameters of the plasmonic resonators are complex, and since the modes lose substantial energy to Ohmic losses and radiate efficiently to the far-field, these lead to low *Q̃* values. Physical quantities such as scattering cross-sections and Purcell enhancements can be constructed from a linear sum of these eigenmodes.^32^ Understanding what influences these modes and how their relative strengths contribute to physical phenomena allows for a holistic understanding of the system. This is especially powerful when there are few QNMs in a spectral region of interest that can dominate the response observed,^33^ but they can also account for systems with many near-degenerate modes.

Here we find the solutions of >2000 QNMs over a variety of different geometric parameters. The results produce general trends that can predict the spectral position and intensity of the first three lowest order QNMs for all NPoM configurations and provide a new benchmark for any further analytical models, as well as optimization conditions for designing nanoplasmonic cavities. Notably, while all modes follow the qualitative trends of past works, they are found to quantitatively vary widely and distinguishably depending on the shape of the nanoparticle facet. Additionally, the effect of facet edge rounding, previously implicated as instrumental in the coupling of light out of the gap^26^ is shown to affect QNMs differently. Finally, the results are compared to experimental measurements of mode positions to allow unique optical identification of the likely facet shape under each NP and its size.

## Results and Discussion

**Figure 1:**
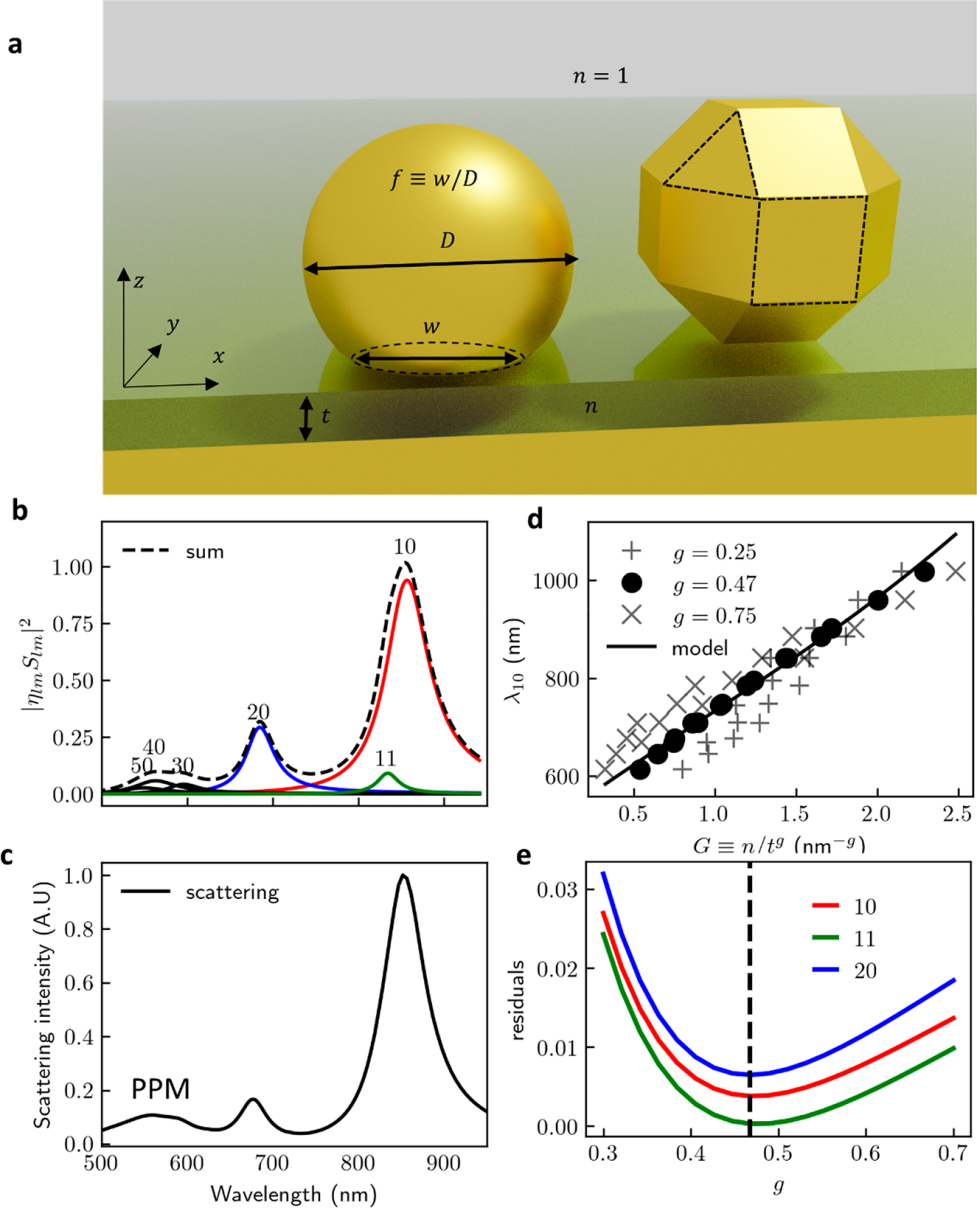
Simulation geometry and plasmon nanocavity modes. (a) Schematic NPoM geometries used, built from Au NP separated from the Au semi-infinite plane by a dielectric gap of thickness *t* and refractive index *n*. (left) Truncated Sphere on Mirror (TSoM) of diameter *D* and circular facet diameter *w*. (right) Rhombicuboctahedron with square and triangular facets highlighted. (b) Spectral response |*S*_*lm*_|^2^ of the six highest-η_*lm*_ QNMs, scaled by η_*lm*_^2^, and their sum (dotted line). The 10, 11, and 20 modes are sufficient to reconstruct the spectral response beyond 600 nm. Higher-order modes, most visibly the 30, 40, and 50 modes, account for the “pseudomode”. (c) Simulated scattering spectrum of TSoM. (d) Wavelength of 10 QNMs for TSoM with *D* = 80 nm and *f* ≡ *w*/*D* = 0.3 vs *G* ≡ *n*/*t*^g^ for three values of *g*. Data become colinear for *g* = 0.47; the line is the third degree polynomial regression fit. (e) Sum of squared residuals of the third degree polynomial fits for λ_10_, λ_11_, and λ_20_ vs *g* for TSoM. Minimal residuals found at average *g* = 0.472 ± 0.004.

We first explore the Truncated Sphere on Mirror (TSoM), which is commonly used to simulate NPoM geometries^34−37^ and consists of a sphere of diameter *D* truncated to produce a circular nanoparticle facet of diameter *w*, separated from a semi-infinite Au substrate by a dielectric gap of thickness *t* and a refractive index *n* inside a surrounding background refractive index of 1 (Figure 1a, left). For a single combination of these parameters, the 145 lowest-energy QNMs of this system are computed by adapting the auxiliary-field eigenvalue formulation of ref (32) using Finite Element Methods and categorized as *lm* by analogy to spherical harmonics *Y*_*l*_^*m*^, with the symmetries of their near-fields extracted at the midplane of the gap. Each computed eigenmode has a characteristic complex frequency ω̃_*lm*_ ≡ ω_*lm*_ – *i*κ_*lm*_ consisting of real and imaginary components. A key feature of QNMs is that they can be used to reproduce the scattering spectrum, that is, *E*_*s*_(ω) = ∑_*lm*_α_*lm*_(ω)*E*_*lm*_, where α_*lm*_(ω) are frequency-dependent scattering coefficients.^32^ These coefficients can be further factored into two parts, α_*lm*_ = *S*_*lm*_(ω)*O*_*lm*_(ω). The former term accounts for the line shape of the mode scattering contribution to each peak at ω = Re[ω̃_*lm*_] and has a width proportional to Im[ω̃_*lm*_], allowing us to reconstruct the scattering spectra (with asymmetries that arise from mode Fano interferences). The latter factor is a near nondispersive term that accounts for the mode coupling to an incident field (see Supporting Information, S1–S2 for a more detailed description). The real part of ω̃_*lm*_ is the spectral position of the QNM, while the imaginary component κ_*lm*_ gives the rate of the total energy loss from the mode (radiative and nonradiative), analogous to the decay rate of an emitter.^32^ The nonradiative decay rate can also be independently calculated from the decay rate κ_MIM_(ω,*n*,*t*) of a similar but nonradiative infinite MIM waveguide, for light at frequency ω = Re[ω̃_*lm*_],^31^ as detailed in Supporting Information, S2. Therefore, for each QNM, the radiative efficiency can be defined as
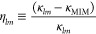
1This radiative efficiency η_*lm*_ is proportional to the scattering intensity of each QNM. The spectral response (|*E*_s_(ω)|^2^) thus takes the form |*S*_*lm*_(ω)|^2^, scaled by the radiative efficiency.

The three most radiative modes (*lm* = 10, 11, and 20) deliver most of the system’s scattering spectral response for λ > 600 nm (Figure 1b,c). As the wavelength approaches the surface plasmon resonance of the isolated Au NP (∼530 nm), the QNMs form a continuum of spectrally overlapping modes that are individually weakly scattering. This plasmon “pseudomode”^38^ is poorly confined within the gap and, thus, is largely uninfluenced by gap morphology, thickness, and refractive index, and instead depends almost entirely on the NP diameter *D*. For normally incident excitation, a lateral dipole/multipole can be excited on the NP as a superposition of *l*1 modes in this mode-dense region. This linear sum of spectrally overlapping orthogonal QNM modes forms the weak “transverse mode” for the system.

Adding more modes does not significantly alter the spectrum, affirming the power of the QNM approach to understand plasmonic nanoantennae through using a few dominant modes in the spectral region of interest. For most NPoM applications, these are the 10, 11, and 20 modes, which are thus the focus here. Dependences of higher-order modes, such as the 21 and 22 modes are presented in Supporting Information, Figure S2 where calculated.

A parametric sweep is performed for the TSoM geometry using all combinations of the parameters shown in Table 1, extracting lowest-energy QNMs for each combination of values. When the calculated mode wavelengths λ_*lm*_ are plotted against the scaled gap parameter *G* ≡ *n*/*t*^g^, they always become collinear for *g* = 0.47 (demonstrated for the 10 mode in Figure 1d with *f* = 0.3, *D* = 80 nm). This shows that the dimensionality of the problem can be reduced and that gap refractive index and thickness cannot be distinguished.

**Table 1:** Parameter Sweeps^a^

parameter	set of values				unit
*f* = *w*/*D*	0.15	0.3	0.46	0.6	
*D*, diameter	40	60	80	100	nm
*t*, gap size	0.75	1.5	3.0	6.0	nm
*n*, refractive index	1.25	1.5	1.75	2.0	

a *f* ≡ *w*/*D* is the relative facet size with respect to the nanoparticle diameter, *D* is nanoparticle diameter, *t* is gap thickness, and *n* is gap refractive index.

Instead of scaling with optical path 2*nDf* (as for microcavities or interferometers), these metal–insulator–metal waveguides support a plasmon with λ_eff_ = π*t*|Reε_*m*_|/*n*^2^ for small *t*. Using a Drude model ε_*m*_ = ε_∞_ – λ^2^/λ_p_^2^ gives the quasi-analytical formula for 10 mode wavelength described in,^20^
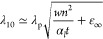
2showing that indeed a dependence as *n*/*t*^0.5^ is expected beyond λ > 600 nm. Clearly, this simple 1D model has to be corrected for 2D confinement, noncircular facets, and leakage beyond the facet edges, but gives a good intuition of the full simulations.

Performing third degree polynomial regression (see Supporting Information, S5–S6) on λ_10_, λ_11_, and λ_20_ with *f*, *D*, and *G* as regressors, a minimum residual is found when *g* = 0.472 ± 0.004. Similar behavior is found for square and triangular facets of the rhombicuboctahedral geometry (described below), resulting in minima at *g* = 0.467 ± 0.006 and 0.465 ± 0.006, respectively. The average of all these, *g* = 0.47, is used throughout the rest of this work. A key conclusion here is thus that the gap refractive index and thickness cannot be independently extracted from spectroscopy.

To investigate the effect of facet shape on the QNMs of NPoMs, a rhombicuboctahedral NP is used since it is one of several common Au nanoparticle shapes observed^35^ and has either the triangular or square facets (outlined in Figure 1a) assembled on the mirror. The facet sizes are increased by slicing the volume (similarly as for TSoMs, although slightly truncating the facet corners) and decreased by extending the faces adjacent to the facet. The side-length and facet side length are defined to preserve the cross-sectional area and ratio of cross-sectional area to facet area (respectively) of that of a TSoM defined by *f* and *D*. For a given *f* or *D*, the area of a circular, square, or triangular facet is thus the same, as well as the NP cross-sectional area. A “regular” rhombicuboctahedron (unaltered bottom facet) has regular facet fraction *f*_*r*_ ≃ 0.3 for the triangular facet, while for the square facet, *f*_*r*_ ≃ 0.46 (see Supporting Information, S3).

**Figure 2:**
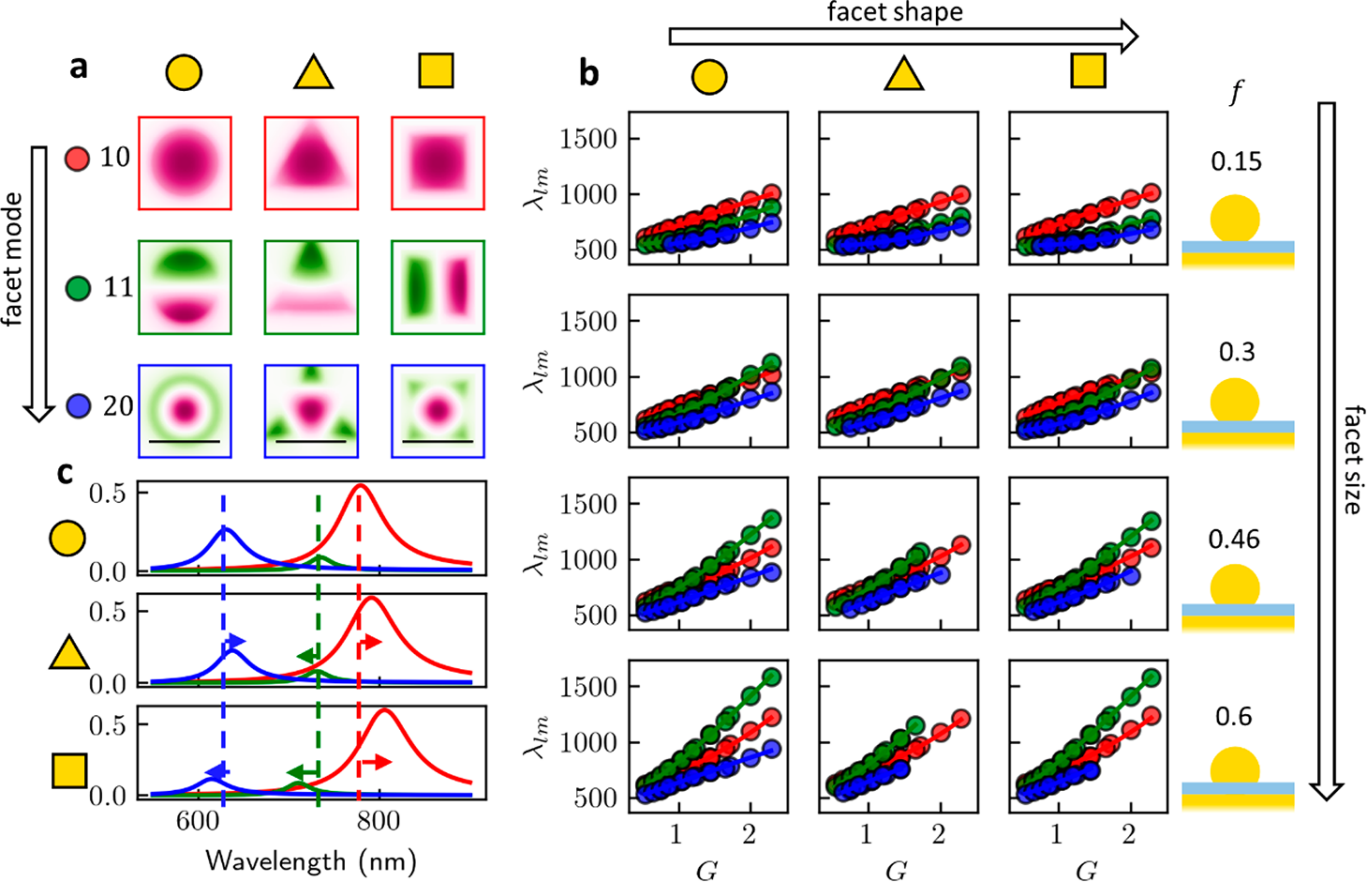
Effect of facet shape and size. (a) Near-field *E*_*z*_ extracted in the middle of the gap for 10, 11, and 20 modes (rows), for circular, triangular, and square facets (columns), for *D* = 80 nm, *f* = 0.3, *t* = 1.5 nm, *n* = 1.5. Scale bar on bottom row is 25 nm. All facets have the same area. (b) QNM wavelength λ_*lm*_ (in nm) for each facet shape (columns), facet fraction (rows), and mode (color), for the *D* = 80 nm subset of the simulated parameter space. Solid lines are polynomial regression fits to the full parameter space. (c) QNM scattering response for 10, 11, and 20 modes (red, green, blue) of circular, triangular, and square facets for the same parameters.

The near-field *E*_*z*_ maps of 10, 11, and 20 modes (Figure 2a) show how the electric field profiles in the gap change with the NP facet shape. As rotational symmetry is still preserved, the two 11 modes remain always degenerate and orthogonal, so the orientation of its nodal line in the near-field is arbitrary (a new pair of 11 modes can be constructed from a sum of any previous orthogonal pair). As most of the electric field is confined within the facet center for the 10 mode, it is relatively unperturbed by gap morphology. The 11 modes however are located near the edges, implying that as facet size *f* increases, they are more perturbed, red-shifting their resonant frequencies. The presence of a radial antinode in the 20 mode near the edge of the facet also suggests it has a strong dependence on both *f* and *G*, since whether this node is within the gap affects its behavior strongly.

For each geometry and combination of parameters in Table 1, the lowest energy QNMs are extracted. Using polynomial regression, the spectral positions are found to be very well predicted with low-order polynomials for the 10 mode with a circular facet. While the 10 mode is well predicted using second degree polynomials, higher-order modes require third degree regression. Polynomials for each geometry and mode are provided in Supporting Information, S5–S6 and are a key result of this work. The 10 and 20 modes tune far less with *f* than *m* ≠ 0 modes, particularly 11. For this reason, identifying the 11 position is highly desirable to determine facet size *f* spectroscopically (see below).

The *D* = 80 nm subset of this data with polynomial fits (Figure 2b) shows the expected trends of mode wavelength increasing with *f*, *D*, and *n*, and decreasing with *t*. The λ_11_ resonance increases more rapidly with increasing *f* than λ_20_, which in turn increases more rapidly than λ_10_, as implied by their near-field profiles (as discussed above). Higher-order modes, such as 22 and 33, whose near-field profiles resemble 2D whispering gallery modes and are thus localized at the facet edges, also redshift disproportionately quickly with increasing *f*, *D*. In fact, for large portions of the parameter space, we find that λ_11_ > λ_22_ > λ_10_ (see Supporting Information Figure S3). Subtle changes in λ_*lm*_ between the three facet shapes can be observed, for instance with *D* = 80nm, *f* = 0.3, *t* = 1.5nm, *n* = 1.5 (Figure 2c). Arrows show how the dominant modes shift differently compared to the TSoM as the facet shape changes (despite the imposed facet area conservation), delivering “shape fingerprints” in the NPoM scattering spectrum (see below).

**Figure 3:**
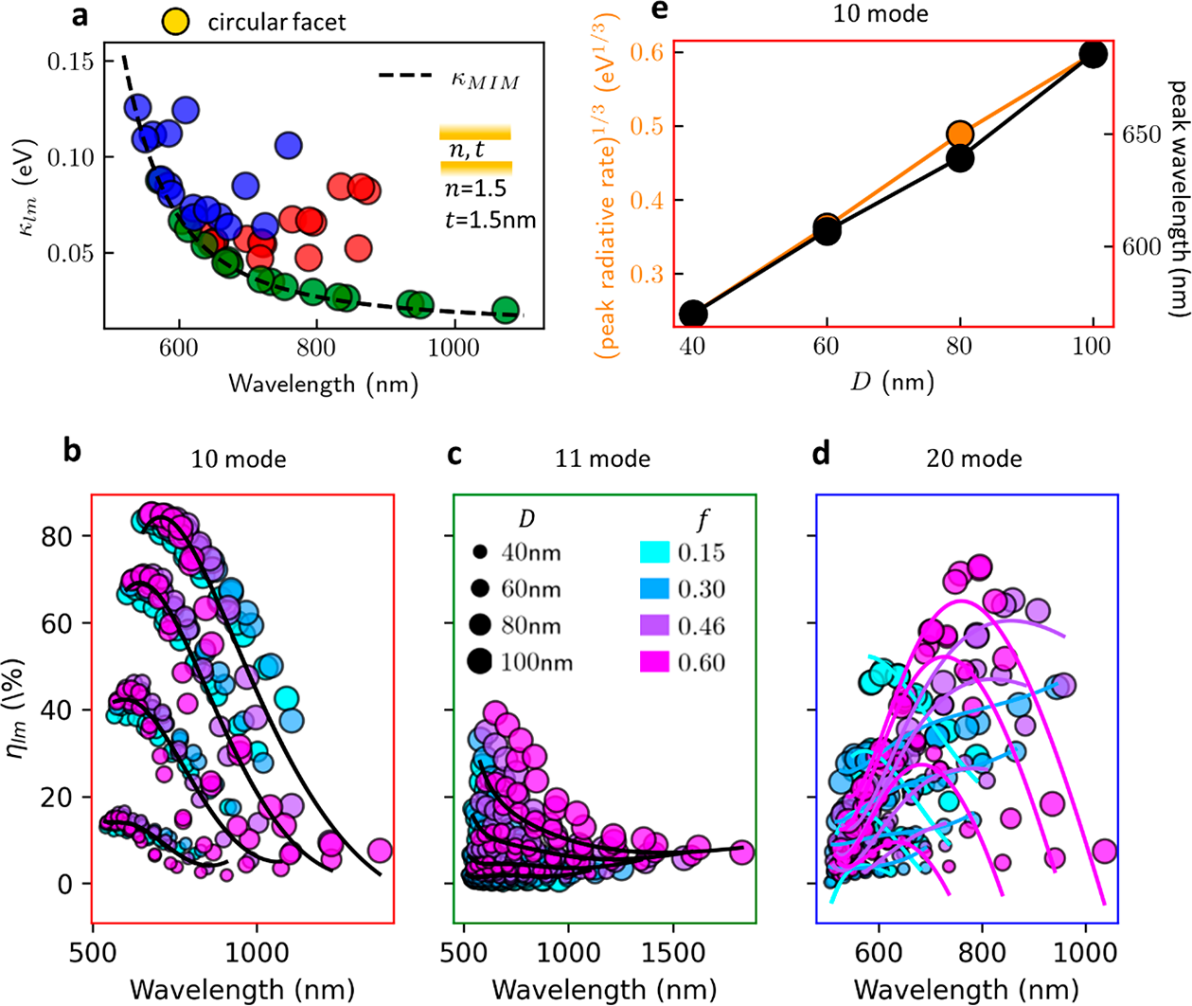
Radiative efficiency of modes. (a) Imaginary component (κ_*lm*_) of QNM frequency vs λ_*lm*_ for *n* = 1.5, *t* = 1.5 nm QNMs, together with κ_MIM_(λ, *n*, *t*) of nonradiative MIM waveguide dispersion (for same gap parameters, dashed line). Only 10 (red) and 20 (blue) modes are radiative (lie above line) compared to 11 mode (green). (b–d) Radiative efficiency η_*lm*_ vs λ_*lm*_ of (b) 10, (c) 11, and (d) 20 modes for circular facet. Black lines show polynomial regressor using *D*, λ_*lm*_, colored lines for η_20_ also incorporate *f*. Crosses in (b) show radiative peak λ_ant_. (e) Cubed root of peak radiative rate vs NP diameter *D* (orange) demonstrating *D*^3^ antenna scaling and peak radiative wavelength λ_ant_ for 10 mode from (b) vs *D*.

The radiative efficiency η_*lm*_ depends on *f*, *D*, *t*, and *n* in a very systematic way. Comparing the imaginary versus real parts of the complex mode frequencies ω̃_*lm*_ (loss vs tuning, Figure 3a) with the nonradiative MIM mode (dashed line) for the same parameters shows how it matches the nonradiative modes. Using eq 2 thus allows the radiative efficiencies to be compared across modes. We see that η_10_ depends only on *D* and λ_10_ (Figure 3b) and is more emissive than the higher-order modes (Figure 3c,d). For each *D*, the 10 mode radiates best at a specific wavelength λ_ant_, corresponding to the antenna response of the system. This antenna radiative rate scales ∝ *D*^3^, as expected from the NP polarizability. The antenna frequency regime is consistent with previous analytical models analyzing the NPoM in terms of its equivalent circuit (Figure 3e)^24^ (see Supporting Information, Figure S5). The weak dependence on *f* is likely due to the decreasing total volume of the NP when truncation increases.

When the 10 plasmonic nanocavity mode frequency coincides with this antenna frequency, scattering is maximized. The analysis also shows that the 11 mode becomes more radiative for large *t* and small *n* (small *G*) at short wavelengths (Figure 3c), because the 11 mode near-field then extends outside of the facet area. In these circumstances, it couples more effectively to excitation fields, radiating vertically (normal to the mirror), however when the 11 mode is within the facet area, this vertical radiation is suppressed. The behavior of η_20_ is strongly affected by facet fraction *f* as well as *D*, λ_*lm*_. This is due to the proximity of the radial antinode with the facet edge, with the radiative efficiency becoming significantly poorer when this antinode encounters the facet edge itself. This becomes clearer when separating η_20_ for each *D* comparing the circular, square, and triangular facets (Supporting Information, Figure S6). These observations suggest that the facet edges can be highly important in selecting which modes are possible to couple to. We thus now explore a further geometrical parameter, the rounding of this facet edge.

**Figure 4:**
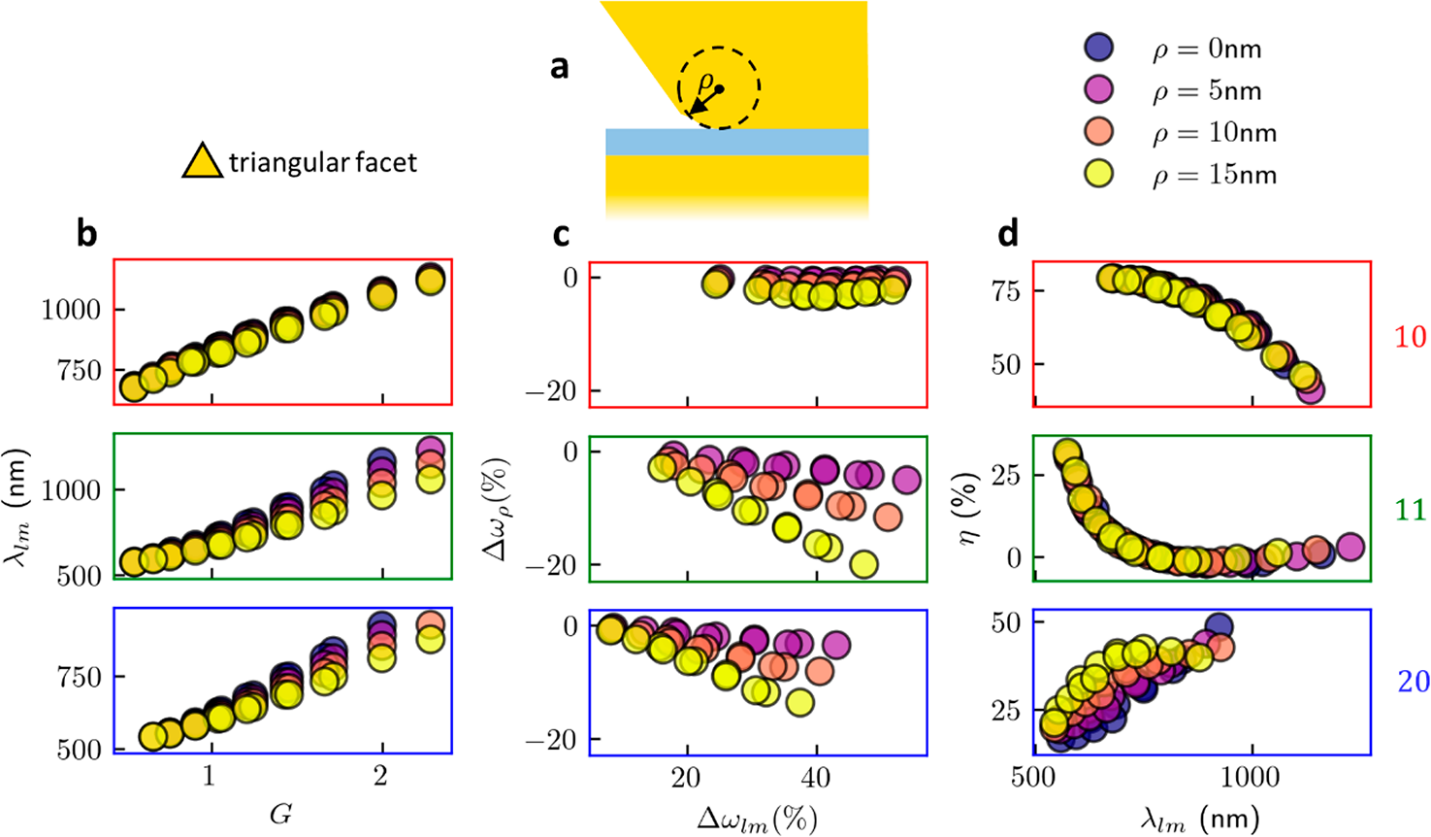
Effect of rounding the facet edge. (a) Schematic of facet rounding parameter ρ, the radius used to fillet the bottom facet. (b) λ_*lm*_ vs gap coupling parameter *G* for *lm* = 10, 11, and 20 (red, green, and blue bordered plots), *D* = 80 nm, *f* ≃ 0.3. Edge rounding has little effect on 10 mode. (c) Fractional frequency shift of ω_*lm*_ due to edge rounding Δω_ρ_ ≡ (ω_*lm*,ρ=ρ_ – ω_*lm*,ρ=0_)/ω_*lm*,ρ=0_, plotted against fractional frequency shift of ω_*lm*_ from the isolated NP resonance, Δω_*lm*_ ≡ (ω_NP_ – ω_*lm*,ρ=ρ_)/ω_NP_, highlighting how the effect of rounding is proportional to the coupling to the mirror. (d) η_*lm*_ vs λ_*lm*_ shows little dependence of radiative coupling on edge rounding.

The rounding radius ρ of the filleted NP bottom (triangular) facet edge is varied (*D* = 80 nm, *f* ≃ 0.3). For all modes, the effect of rounding increases as gap parameter *G* increases (tighter optical confinement, Figure 4). The effect of edge rounding is found to be proportional to the frequency difference Δω_*lm*_ between the isolated NP resonance and the NPoM *lm* plasmon mode. In all cases, rounding the edges blueshifts the modes from the unrounded case (Δω_ρ_) because the effective size of the facet is decreased. This blue-shifting increases with *G* since the modes are more strongly localized under the facet edge, increasing its influence. This effect is stronger for the 11 and 20 modes, as their fields are more localized at the facet edge, with Δω_11_ > 20% at ρ = 10 nm for high *G*. By contrast, for the 10 mode at high *G*, Δω_ρ_ decreases because the high confinement causes the 10 mode profile to retract from the facet edges, outcompeting any rounding effect. The shifts and relative magnitudes caused by increasing ρ correlate with the dependence on decreasing *f*, Δω_11_ > Δω_20_ > Δω_10_, indicating that the dominant effect of increasing ρ is decreasing the facet area and, as such, is well accounted for by the facet size *f*.

The change in radiative efficiency is found to be small for the 10 and 11 modes (Figure 4d). This implies that intuitive ideas based on the nanocavity plasmon *E* field leaking around the facet edge depending on its rounding are incorrect. Previous work suggested that the facet edge angle was also important^26^ but is not evidenced for the 10 mode (Figure 3b). There is, however, a stronger effect on the 20 mode, as the radial antinode is near the facet edge.

**Figure 5:**
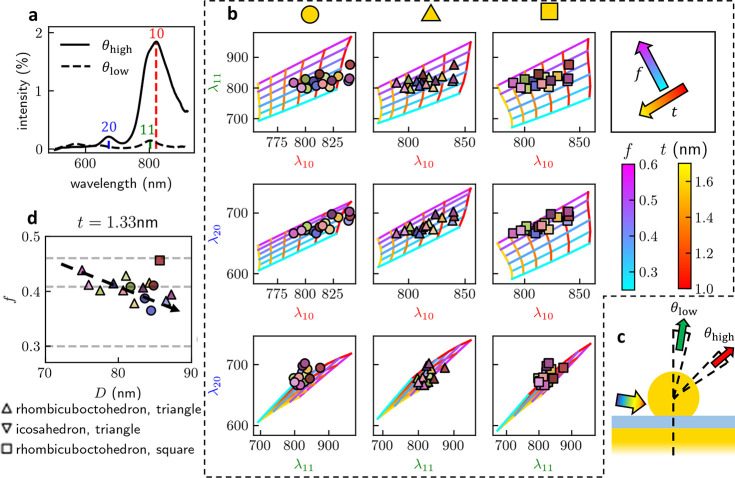
Fingerprinting experimental spectra. (a) Experimental white-light high-angle (θ_high_) and low-angle (θ_low_) single NPoM scattering spectra. Mode positions of 10 and 20 are extracted from former, 11 from latter. (b) Correlations between λ_*lm*_ for 10, 11, and 20 modes plotted for 17 measured NPoMs. For each facet geometry (columns), the relationship between each mode pair is mapped from polynomial fits: plotted vs *t* for a range of *f* (blue-purple lines) and vice versa (red-yellow lines). The three correlations should identify similar (*f*, *t*), and best agreement is seen for triangular facets (center column). (c) Schematic of high- and low-angle collection of scattering. (d) Most-probable facet shape (marker shape) for each NP vs their extracted *f*, *D*. Horizontal lines correspond to the facet sizes *f* for platonic NP shapes, indicating many NPs are likely triangular faceted with *f* ≃ 0.4. This corresponds to either annealed rhombicuboctahedron facets, or perfect icosahedron facets.

High-angle (θ_high_) and low-angle (θ_low_) white-light scattering spectra are taken of 17 NPoMs with a biphenylthiol (BPT) gap spacer molecular layer (*n* ∼ 1.5, *t* ∼ 1.3 nm) by angularly separating the collected light (see Methods). A typical example (Figure 5a) shows that, as expected, the 10 and 20 modes radiate primarily at high angles, while the 11 mode radiates along the normal to the mirror, as previously shown in NPoMs.^20^ This angular decomposition separates the ∼800 nm spectral peak into the overlapping 10 and 11 modes, which we show is highly desirable for assigning a facet shape.

For visualization purposes, the refractive index *n* is fixed at 1.45 and NP diameter *D* at 82 nm (see below). Changing *D* tunes all modes similarly within reasonable polydispersities (80 ± 10 nm) of our samples and thus has little explanatory power (see Supporting Information, Figure S7a). While *n* and *t* may be combined without loss of information into the gap coupling parameter *G* (Figure 1d), here we use *t* (nm) for easy comprehension. To visualize the information in the cross-correlations of the three measured mode positions, the polynomial fits of the 10, 11, and 20 mode wavelengths for each facet shape are plotted on 10 versus 11, 10 versus 20, and 11 versus 20 graphs (Figure 5b). In each, we fix the facet *f* and vary gap size *t* (and vice versa), allowing the set of each NPoM peaks to unambiguously predict a value of (*f*, *t*) for each geometry. The agreement between (*f*, *t*) for these three correlations is a measure of how likely this simulated facet shape is the one probed in experiment. The data points coincide at similar parameter values for the triangular facet (Figure 5b, center column), suggesting most NPoMs have triangular Au(111) facets facing down.

By allowing free variation of *f*, *D*, and *t* and minimizing the residuals across the different facet shapes (see Supporting Information, Figure S5b), we find that most NPoMs have triangular facets, with average *D̅* = 82 ± 5 nm and *t̅* = 1.4 ± 0.3 nm. This *D̅* matches well with the expected 80 nm NP size. Previous ellipsometry measurements suggested that similarly prepared BPT SAMs have thickness *t* = 1.3 nm and refractive index *n* = 1.45,^39^ agreeing well with these optimized parameters.

Since the gaps are well defined in these robust samples, *G* ≡ *n*/*t*^0.47^ does not vary from NPoM to NPoM, and *n* and *t* are thus fixed to investigate the correlation of *f* and *D* (Figure 5d). Triangular-faceted NPoMs (12 out of the 17) have *f* clustered around 0.4–0.5, indicating that their facets are relatively large (the circular facets are rather similar). This might be attributed to facet growth/annealing of rhombicuboctahedral facets (regular rhomboctahedra have *f*_*r*_ ≃ 0.3), however, it is more likely from icosahedra which consist entirely of triangular facets for which *f*_*r*_ = 0.408 or cuboctahedra for which *f*_*r*_ = 0.47. This accounts well for the prevalence of triangular facets assigned. Typically, all these shapes of particles can be identified in electron microscope images, as well as less frequent pentagonal bipyramids.^35,40^ The inverse correlation between *f* and *D* (dashed arrow Figure 5d) is also expected, as faceting generally increases with decreasing metal radius, with surface energy terms becoming more relevant. The full analysis of the three lowest wavelength plasmons observed in the NPoMs thus allows us to extract detailed parameters for their nanoscale morphologies.

A key highlight here is thus the experimental confirmation of facet size and shape based on theoretical simulations (provided in an online tool) and experimental spectra. These have previously been causing confusion in many experimental papers from diverse researchers. Despite predictions of our analytic analysis, the gap scaling here was not seen in previous simulations because they were not accurate enough, while not being obvious to any intuition.

## Conclusion

We show how 3D morphology influences plasmonic nanocavity modes. By employing improved computational methods, detailed information can be now extracted about heterogeneous ensembles on a construct-by-construct basis. More specifically, we show that a handful of QNMs dominate the spectral response of NPoMs, and these tune widely with geometrical parameters. Their resonant frequency dependence on the gap parameters (refractive index *n* and width *t*) can be described entirely in terms of a composite gap parameter *G* ≡ *n*/*t*^0.47^ and low-order polynomials of *D*, *f*, and *G*. The radiative efficiency of the dominant mode scales in intensity and spectral position as for a classical antenna, depending only on spectral position and *D*. Finally, angle-dependent spectroscopy isolates “dark” modes in experimental scattering spectra from NPoMs. Using the lowest three modes in comparison with simulated results allows likely facet shapes to be assigned as mostly triangular. The insights from this analysis support a wide range of experiments, which employ ultralow volume plasmonic cavities, providing their mode spectrum and its sensitivity to geometry at the nm-scale. This underpins a wide variety of applications that utilize these nanocavities.

## Online App

To aid the reader, we provide an online app (see https://www.np.phy.cam.ac.uk/npom-calculator), which gives the mode positions and estimated scattering spectra for any combination of the above parameters, as well as refer to the full parameter sets in the Supporting Information. Since the three-dimensional nanoparticle shape above the facet has much less effect on the modes (mainly the height controls the antenna resonance λ_ant_), this model works for every typical NPoM shape. The source python code is freely accessible. We note that the same QNMs are also found in nanoparticle dimers, but experimentally the overlap of the two touching facets is uncontrolled, leading to very widely heterogeneous tuning ranges compared to the NPoM (as expected from the influence of above).

## Methods

Finite Element Method simulations were performed with COMSOL adapting the QNMEig toolkit.^24^ A multipole Lorentz–Drude model was used to model ε_Au_(ω), necessary for the augmented-field formulation of QNM decomposition.
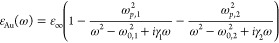
ε_∞_ = 6, ε_0_, ω_*p*,1_ = 5.37 × 10^15^ rad/s, ω_0,1_ = 0 rad/s, γ_1_ = 6.216 × 10^13^ rad/s, ω_*p*,2_ = 2.636 × 10^15^ rad/s, ω_0,2_ = 4.572 × 10^15^ rad/s, and γ_2_ = 1.332 × 10^15^ rad/s. These parameters were obtained by fitting to Johnson and Christy Au.^41^ This data and the fit are presented in Supporting Information, S10. Where background fields are required, a TM plane wave incident on the same structure without the NP is simulated with periodic boundary conditions.

All simulated geometries have 5 nm rounding applied to the bottom facet only. A quarter geometry and appropriate symmetry was used to reduce the computational time for TSoMs. With accurate search regions for the QNMs and careful choice of meshing, the full set of simulations takes a week with 15 cores on COMSOL.

NPoM samples were prepared on template-stripped Au, fabricated by established methods.^42^ A biphenyl-4-thiol (BPT; Sigma-Aldrich, 97%) SAM was formed by submerging the substrate in a 1 mM solution in anhydrous ethanol (Sigma-Aldrich, <0.003% H_2_O) for 24 h, then rinsing with ethanol. The 80 nm Au NPs (BBI solutions) were then dropcast on the surface.

Individual NPoMs are illuminated with focused incoherent white light (halogen lamp) at an annular illumination angle of 64–75° with respect to normal incidence, and scattered light with an angle of <64° is collected through a dark-field objective (Olympus 100x BD, NA 0.9). The scattering pattern from a NPoM is determined using the light intensity distribution at the back focal plane of the microscope objective. Single NPoM structures are spatially isolated by spatially filtering the real image plane with a pinhole. The back focal plane image is demagnified by three times before being imaged on the entrance slit (150 μm wide) of a Triax 320 spectrometer, where a narrow range of the scattering pattern near *k_x_*/k_0_ = 0 is filtered and dispersed by a 150 l/mm grating and collected using an Andor Newton 970 BVF EMCCD.

## Supporting Information Available

Relating QNMs to scattering: Details of how QNM complex frequencies can be related to scattering intensity. Radiative efficiency of QNMs: Its motivation, definition, and description. Simulation geometry: Exact details of the simulation geometry, in particular, the NP facet. QNM wavelengths mapped across parameter space: All simulated QNMs and their polynomial fits. Polynomial regression terms: Information necessary to reconstruct the polynomial fits for oneself. Influence of *f* on QNMs: Exploration of how the facet size affects modes differently. Radiative efficiencies for each facet geometry: All radiative efficiencies and their fits. Assigning facet shapes and extracting *f*, *D*, *t*: Schematics detailing the exact procedure for assigning an angle-resolved darkfield spectrum to a NP facet shape. Fit of Au permittivity: Multipole Drude–Lorentz fit of Au used throughout this work (PDF)
