# Supplementary material for: Fingerprinting the Hidden Facets of Plasmonic Nanocavities
Source: ACS Photonics. 2022 Jul 27;9(8):2643–51. doi: 10.1021/acsphotonics.2c00116 (PMC9389613; doi:10.1021/acsphotonics.2c00116)
Supplement: Supplementary file 1 — ph2c00116_si_001.pdf [file ph2c00116_si_001.pdf]

Supporting Information:

Fingerprinting the Hidden Facets of Plasmonic Nanocavities

Eoin Elliott[1], Kalun Bedingfield[2], Junyang Huang[1], Shu Hu[1], Bart de Nijs[1],
Angela Demetriadou[2*], Jeremy J Baumberg[1*]

[1] *NanoPhotonics Centre, Cavendish Laboratory, University of Cambridge, Cambridge CB3 0HE, UK.*

[2] *School of Physics and Astronomy, University of Birmingham, Edgbaston, Birmingham, B15 2TT, UK.*

Number of pages: 10

Number of figures: 7

Number of tables: 4

- **Relating QNMs to scattering**
- **Radiative efficiency of QNMs**
- **Simulation Geometry**
- **QNM wavelengths mapped across parameter space**
- **Polynomial regression terms**
- **Influence of f on QNMs**
- **Radiative efficiencies for each facet geometry**
- **Assigning facet shapes and extracting f, D, t**
- **Fit of Au permittivity**

Relating QNMs to scattering

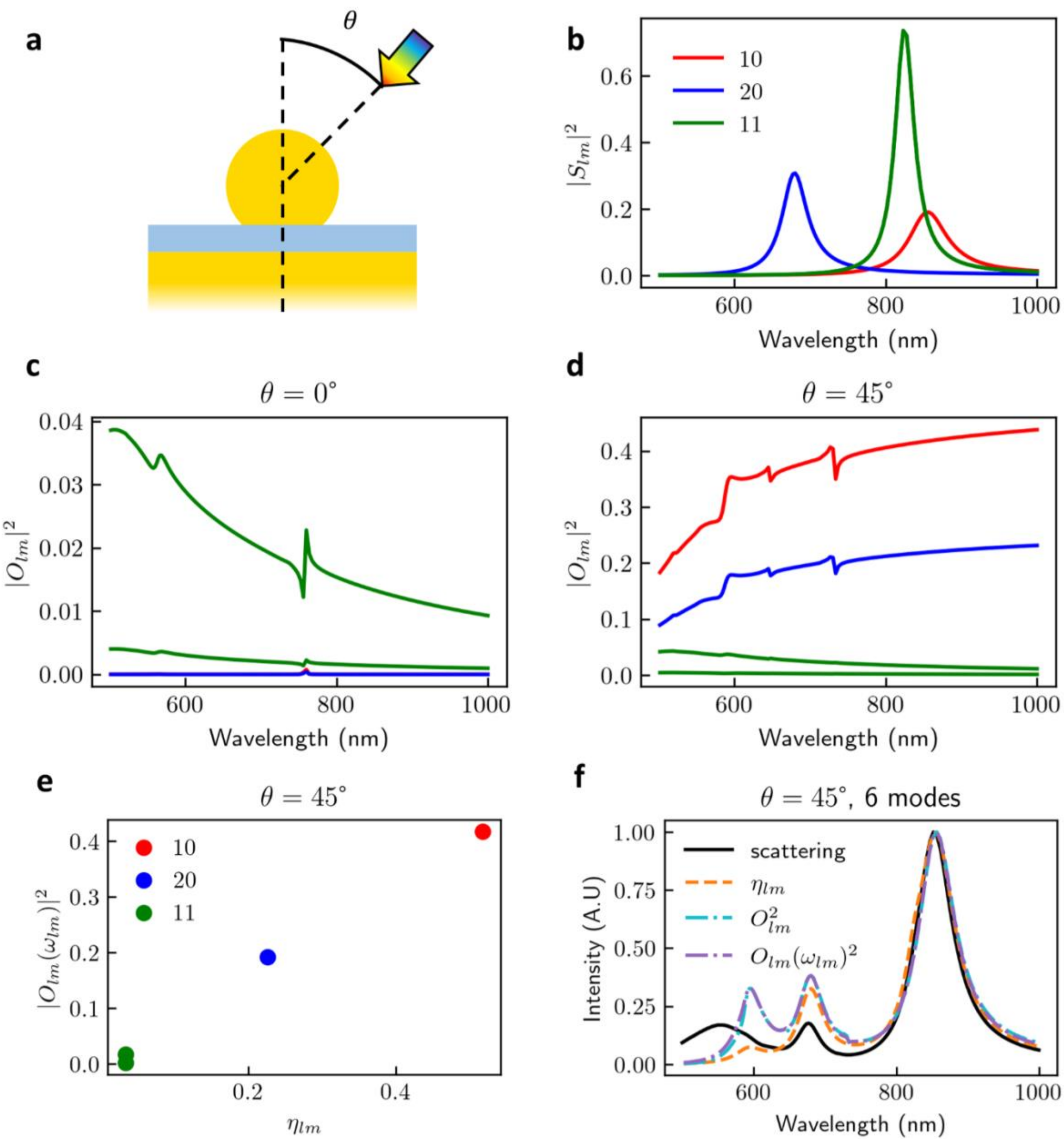

Figure. S1. a, Definition of incident angle (θ) of background E fields. **b,** Lineshapes S_{lm} of $10, 20, 11$ modes, without scaling. **c-d,** Overlap O_{lm} evaluated for 0,45° background fields. **e,** Comparison of $|O_{lm}(\omega_{lm})|^2$ and radiative efficiency η. **f,** Reconstructing the scattering spectrum of TSoMs using scaling of QNMs by multiplying S_{lm} by η_{lm}, $O_{lm}(\omega)^2$ and $O_{lm}(\omega_{lm})^2$.

Quasi-normal modes (QNMs) can be used to construct the scattering spectrum [Ref 32, main text], i.e. $E_s(\omega) = \sum_{lm} \alpha_{lm}(\omega)\, E_{lm}$. The permittivity of Au can be described with a multipole Lorentz-Drude model,

$$\varepsilon_{Au}(\omega) = \varepsilon_\infty \left(1 - \frac{\omega_{p,1}^2}{\omega^2 - \omega_{0,1}^2 + i\gamma_1\omega} - \frac{\omega_{p,2}^2}{\omega^2 - \omega_{0,2}^2 + i\gamma_2\omega}\right)$$

where $\varepsilon_\infty = 6, \varepsilon_0$, $\omega_{p,1} = 5.37 \times 10^{15}$rad/s, $\omega_{0,1} = 0$rad/s, $\gamma_1 = 6.216 \times 10^{13}$ rad/s, $\omega_{p,2} = 2.636 \times 10^{15}$ rad/s, $\omega_{0,2} = 4.572 \times 10^{15}$ rad/s, and $\gamma_2 = 1.332 \times 10^{15}$ rad/s. With this, and defining for convenience the term $\Delta\epsilon(\omega) = \epsilon_{Au}(\omega) - \epsilon_b$ where ϵ_b is the relative permittivity of the background medium (in this case air), the scattering terms $\alpha_{lm}(\omega)$ take the form

$$\alpha_{lm}(\omega) = \left[\varepsilon_b - \varepsilon_\infty - \frac{\tilde{\omega}_{lm}}{\omega - \tilde{\omega}_{lm}} \Delta\varepsilon(\tilde{\omega}_{lm})\right] O_{lm}(\omega) \equiv S_{lm}(\omega) O_{lm}(\omega), \tag{SI.1}$$

where $O_{lm}(\omega) = \sum_{lm} E_b(\omega) \cdot E_{lm}$ is the overlap integral between an excitation (or 'background') field E_b (propagating in free space and reflecting off the mirror) and the QNM field E_{lm} over the resonator volume V_r (in this case the NP) [Ref 32, main text], and which represents the 'coupling' between the QNM and the excitation field. The first term dominates the lineshape of each mode, as $O_{lm}(\omega)$ varies little across the linewidth of each QNM (see SI Fig. S1c-d) and so $O_{lm}(\omega) \simeq O_{lm}(\omega_{lm}) \equiv O_{lm}$. The magnitude of O_{lm} accounts for the difference in scattering cross-section of vertically-oriented dipole-like $l0$ modes which scatter E_z fields strongly, and 11, 21 modes which only weakly scatter $E_{x,y}$ fields. To compute O_{lm} for multiple type QNMs, one only needs the excitation fields $E_b(\omega)$ in the absence of the NP, and the QNM field E_{lm}, demonstrated for a single geometry in Supporting Information Figure S1c-d. The radiative efficiency η_{lm} is found to be proportional to $|O_{lm}|^2$ (shown in Supporting Information Figure S1e for 45° angle of incidence). This efficiency η_{lm} does not depend on the choice of incident field (as for O_{lm}) or the position of a dipole within the gap (as with figures of merit based on the Purcell enhancement factor) but is instead a quantity inherent to each QNM. The near-field scattered intensity $|E_s|^2$ is obtained using $|\eta_{lm} S_{lm}(\omega)|^2$ (as used in Fig. 1b,c) or $\left|O_{lm}^2(\omega) S_{lm}(\omega)\right|^2$, as shown in SI Fig. S1(f). When summing the mode scattering intensities here a first approximation is made that $|\sum_{lm} E_{s,lm}|^2 \simeq \sum_{lm}\left|E_{s,lm}\right|^2$ which sidesteps mode interference effects. Because the spectrally-close modes we consider generally do not overlap in far-field directions, this will have a small effect on the intensity (but not position) where the modes spectrally overlap.

We first consider Eq. 1 (main text) which expresses the scattered E field as a product of 2 terms, $S_{lm}(\omega)$, and $O_{lm}(\omega)$, which is an overlap integral between a background excitation field and the QNM field within the resonator volume. In the main text Figure S1b shows $|S_{lm}|^2$ (as scattering intensity $\propto |E|^2$) giving the characteristic symmetric lineshapes of QNMs. Their intensity however does not reflect the brightness of the modes. Supporting Information Figure S1c shows $O_{lm}(\omega)^2$, which is much larger for the two 11 modes (green) for normal ($\theta = 0$) incidence, as expected due to the lateral dipole moment of this mode. The difference between the 11 modes is due to the relative orientation of the random ϕ-dependence (but orthogonal) 11 modes on the surface, and the background field polarization. Both sharp resonances and broad background reflections are seen. O_{l0} modes are much stronger than O_{11} modes for $\theta > 0$ (Fig. S1d). In all cases, $O_{lm}(\omega)$ varies little across the linewidth of a QNM, motivating the approximation $O_{lm}(\omega) \simeq O_{lm}(\omega_{lm})$. The parameter η_{lm}, a measure of the radiative efficiency of each mode derived from comparison with a similar MIM structure, is proportional to $|O_{lm}(\omega_{lm})|^2$ (Fig. S1e), as expected since both relate directly to the spectroscopic intensity of the modes. Multiplying $S_{lm}(\omega)$ by either η_{lm} or $O_{lm}(\omega_{lm})^2$ before taking the absolute square is then needed to get the appropriate scattering spectrum (Fig. S1f).

Radiative efficiency of QNMs

A Metal-Insulator-Metal (MIM) waveguide structure consists of semi-infinite metal surfaces separated by an insulator of refractive index n and thickness t and can support gap plasmons with frequency ω_{MIM} and loss κ_{MIM}. The complex plasmon frequency $\tilde{\omega}_{MIM} \equiv \omega_{MIM} - i\kappa_{MIM}$ is known from the parametric equation for the dispersion [Ref 1, main text]

$$\tanh\left(t\sqrt{\beta^2 - (n\tilde{\omega}_{MIM}/c)^2}\right) = -\frac{n^2\sqrt{\beta^2 - (\tilde{\omega}_{MIM}/c\,)^2\epsilon_{Au}(\omega_{MIM})}}{\epsilon_{Au}(\omega_{MIM})\sqrt{\beta^2 - (n\tilde{\omega}_{MIM}/c\,)^2}} \qquad \text{(SI. 2)}$$

for given wavevector β. This is solved for 300 values of β, for 20 values of each n and t and then fitted, yielding $\tilde{\omega}_{MIM}$ which allows the computational extraction of $\kappa_{MIM} = F(\omega_{MIM}, n, t)$. This gives the non-radiative background absorption of the MIM waveguide under the NPoM facets.

Simulation geometry

The parameter D was defined for a rhombicuboctahedron to preserve the cross-sectional area of a sphere of diameter D. The horizontal cross-sectional area of a rhombicuboctohedron of side length a is $2(1+\sqrt{2})a$ (square facet down),

and the sphere cross-section is $\pi(D/2)^2$, leading to $D = a\sqrt{(1+\sqrt{2})\left(\frac{8}{\pi}\right)}$. The facet fraction f is defined for the TSoM as the ratio of the facet diameter to the sphere diameter. For a rhombicuboctohedron, f is defined so as to preserve the ratio of facet area to cross-sectional area, i.e. f^2 is the ratio between cross-sectional area and facet area. The facet area of a NPoM is thus preserved for the same D, f, independently of shape. For a triangular facet of side length b this is $\left(\frac{b}{a}\right)\left(\frac{3^{1/4}}{2\sqrt{2(1+\sqrt{2}}}\right)$, and for a square facet $f = \left(\frac{b}{a}\right)\left(\frac{1}{2(1+\sqrt{2})}\right)$. The facet fractions of regular rhombicuboctahedra are found when $b = a$. The regular facet fraction of an icosahedron and a cuboctohedron is defined similarly.

QNM wavelengths mapped across parameter space

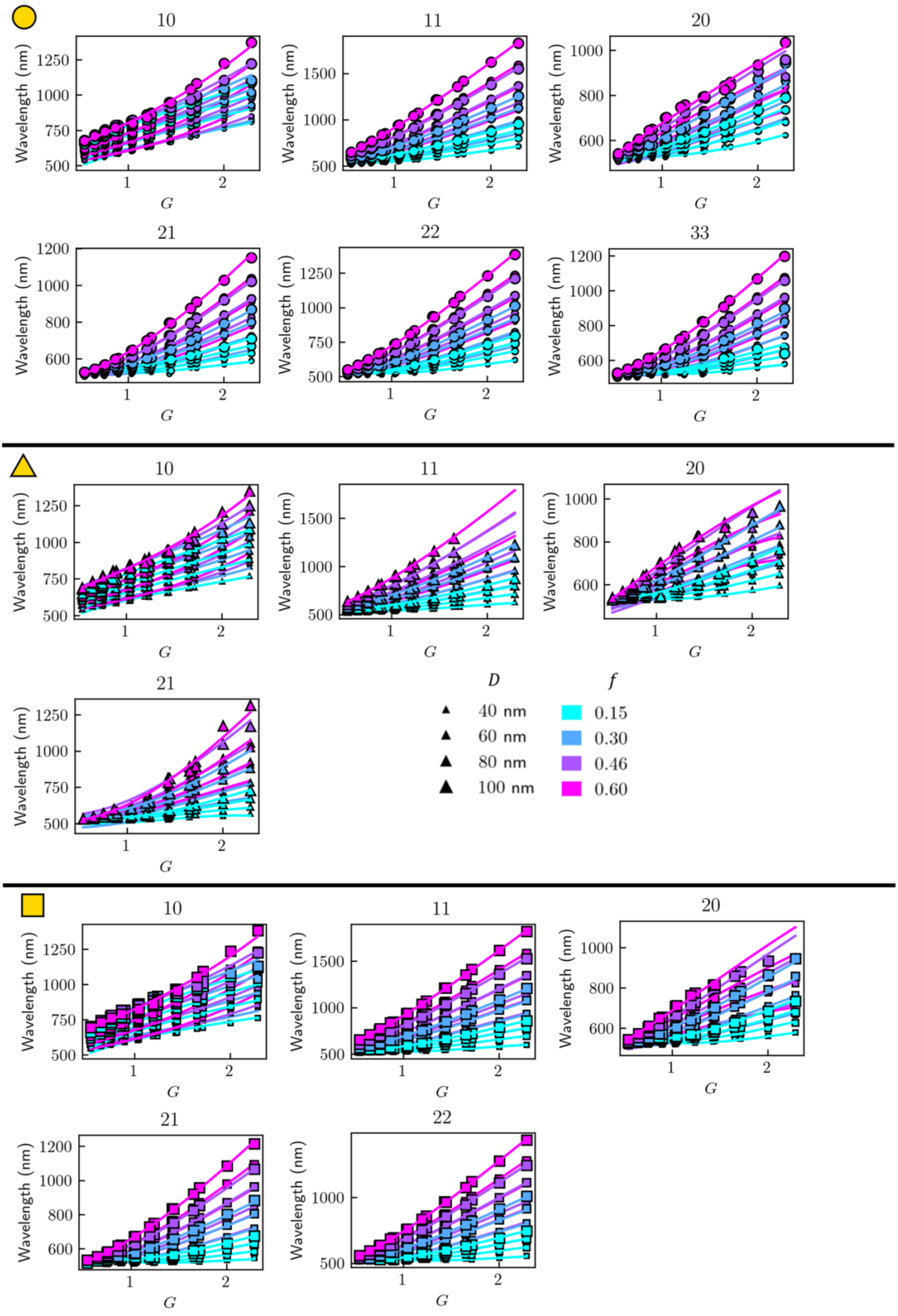

Fig S2 QNM wavelengths for simulated circular, triangular and square facets with polynomial regression (lines).

Polynomial regression terms

Parameter/Term #	1	2	3	4	5	6	7	8	9	10	11	12	13	14	15	16	17	18	19	20
f	0	1	0	0	2	1	1	0	0	0	3	2	2	1	1	1	0	0	0	0
D	0	0	1	0	0	1	0	2	1	0	0	1	0	2	1	0	3	2	1	0
G	0	0	0	1	0	0	1	0	1	2	0	0	1	0	1	2	0	1	2	3

Table S1 | Parameter exponents for each term

Mode/ Term #	1	2	3	4	5	6	7	8	9	10	11	12	13	14	15	16	17	18	19	20
10	195	1480	-0.297	364	-2180	-2.46	-1150	0.0157	2.1	-105	1090	0.379	719	-0.00187	3.31	211	3.99E-05	-0.0146	0.234	8.75
11	666	-341	-0.807	-275	453	-5.49	240	0.00651	1.21	141	-172	2.14	-236	0.0158	8.73	25.5	3.08E-06	-0.00853	0.254	-26.6
20	744	-1090	-1.85	-297	1280	4.05	842	-0.0072	2.77	78.4	-233	-5.13	-719	0.00977	1.19	-68	3.15E-05	-0.00192	-0.31	-4.25
22	622	-222	-0.772	-134	546	-3.57	-88	0.017	-0.362	77.6	-296	0.692	-121	-0.00457	5.85	73.4	-3.59E-05	-0.00547	0.433	-18.8
21	786	-608	-2.56	-369	677	0.554	279	0.00766	1.92	156	-179	-1.66	-269	-0.00342	5.59	29.7	2.03E-05	-0.00886	0.139	-27.8
33	801	-778	-1.81	-354	1200	-0.297	231	0.00184	1.56	143	-444	-2.63	-361	0.000326	5.6	49.7	4.65E-05	-0.00931	0.0676	-25.5

Table S2 | Circular facet term coefficients

Mode/Term #	1	2	3	4	5	6	7	8	9	10	11	12	13	14	15	16	17	18	19	20
10	267	1120	-1.3	309	-2100	2.03	-658	0.0124	3.08	-151	1440	-2.38	270	-0.00513	1.45	200	6.82E-05	-0.0154	0.204	20.3
11	719	-819	-1.95	-230	1070	2.53	443	0.00868	0.61	73.6	-233	-5.04	-609	-0.00304	8.29	85	2.18E-05	-0.00865	0.574	-20.9
20	963	-2070	-3.82	-470	2010	10.5	1630	0.0125	1.94	129	-7.47	-11	-1300	-0.00177	1.38	-168	-4.32E-05	-0.00145	-0.0038	-14.4
21	466	-2070	9.73	24.9	6080	-4.98	381	-0.0709	-6.73	62	-3650	-17.7	-1120	0.0771	7.98	131	1.18E-04	0.0189	1.43	-33.4

Table S3 | Triangular facet term coefficients

Mode/Term #	1	2	3	4	5	6	7	8	9	10	11	12	13	14	15	16	17	18	19	20
10	172	1920	-0.929	330	-3730	-0.183	-964	0.0123	3.4	-135	2400	-0.631	637	-0.00047	1.32	219	6.49E-05	-0.016	0.164	13.9
11	756	-600	-2.07	-367	517	-0.752	461	0.00612	1.7	136	-34.9	-0.117	-396	0.00194	8.27	36.5	3.56E-05	-0.0111	0.354	-28.7
20	1120	-1400	-6.43	-613	1290	6.77	700	0.0277	3.29	197	-242	-4.33	-506	-0.0205	4.51	2.13	-1.84E-05	-0.012	0.00254	-31.7
22	596	-1240	2.91	-51	2530	-3.12	628	-0.0167	-3.36	19.9	-1070	-5.23	-956	0.026	6.86	0.292	-7.24E-06	0.0111	0.809	-9.44
21	863	-816	-4.36	-323	1000	3.52	207	0.0317	1.06	116	-309	-3.5	-294	-0.0238	5.81	56.2	-3.93E-05	-0.0098	0.294	- 23.4
$2-2$	970	-1160	-5.1	-489	1160	6.32	550	0.0206	2.64	164	-275	-4.51	-442	-0.0233	5.57	25.2	9.77E-06	-0.0114	0.123	- 29.6

Table S4 | Square facet term coefficients

The polynomial fits for each λ_{lm} may be constructed term-by-term by raising each parameter to the exponent in Table S1, and taking their product, then multiplying by the coefficients in Tables S2-4, then summing all terms. For example, for a circular facet ($G = n/t^{0.47}$),

$$\lambda_{10}(f, D, G) = 195f^0D^0G^0 + 1480f^1D^0G^0 + \cdots + 8.75f^0D^0G^3$$

Influence of f on QNMs

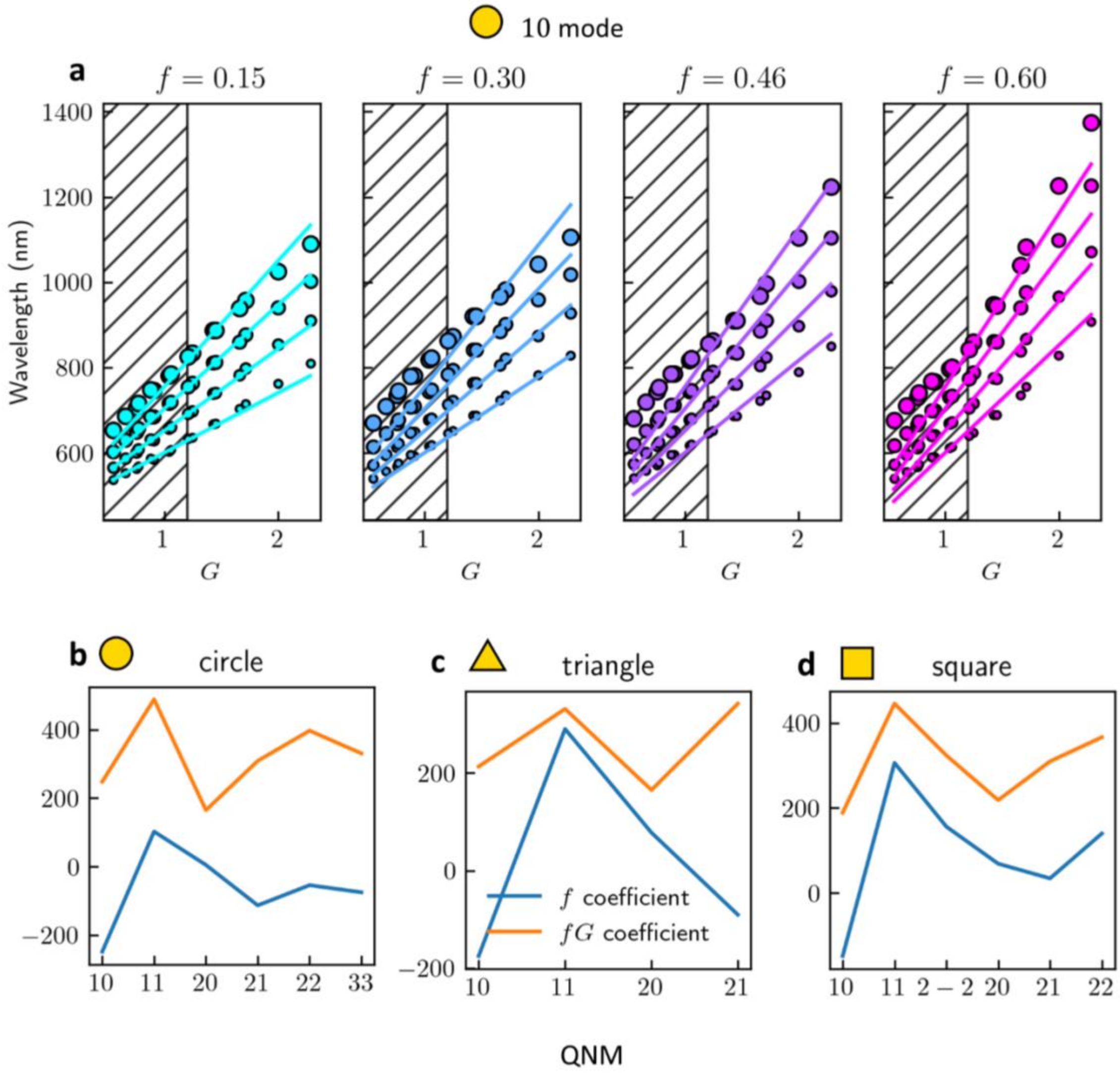

Fig. S3. a, Simplified fits of QNM wavelengths, for $G > 1.2$ using $(1, f, Gf, GD)$ as regressors. **b,** Coefficients of terms with f for each mode and geometry.

We observe that λ_{lm} is linear in G for $G > 1.2$, and thus can be simplified to four terms, $(1, f, Gf, GD)$, which illustrate the different dependencies of f exhibited by different modes. The coefficient of the Gf term indicates how the slope of λ_{lm} vs G varies with f, i.e. measures how each mode lm is affected by the facet. Dependencies of G, f (Fig. S2b, orange) for the 10, 20 modes are smaller than higher-order modes, while 11 mode has the strongest dependence. The f coefficient behaviour (blue) confirms that this is not compensation from this term.

Radiative efficiencies for each facet geometry

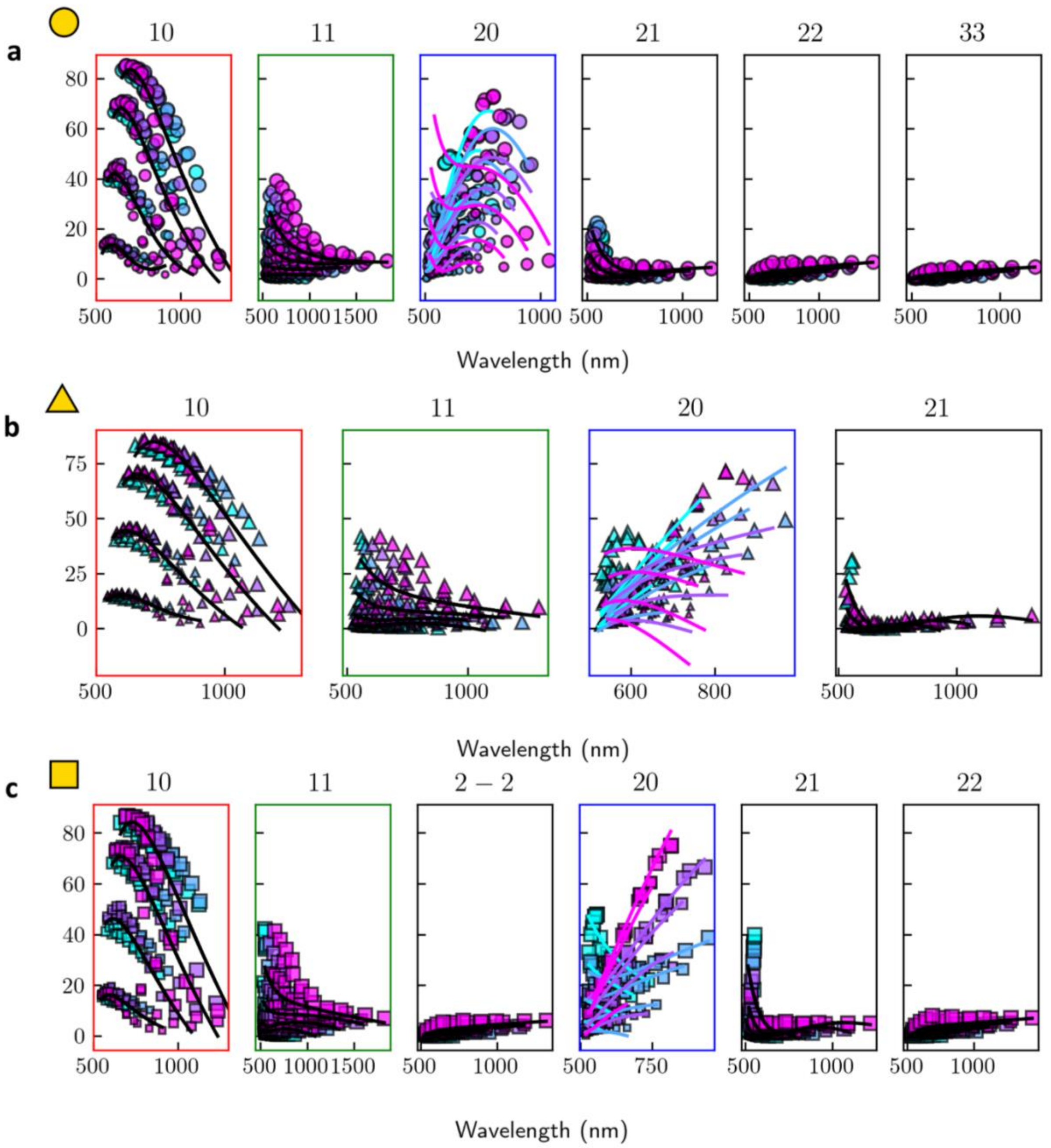

Fig. S4. QNM Radiative efficiencies η_{lm} as a vs QNM wavelength, for circular (a), triangular (b), and square (c) facets.

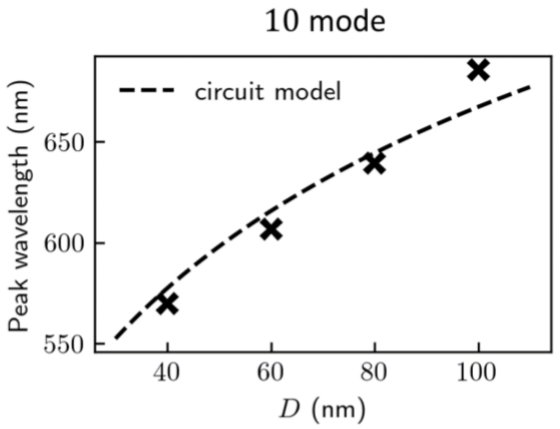

Fig. S5. 10 mode peak (highest-η) wavelength compared with the circuit model for coupled antennae [Ref 24, main text]with parameters $\chi = 0.5$, $\zeta = 0.055$. Peak wavelengths are those illustrated in Fig. 3b.

Assigning facet shapes and extracting f, D, t.

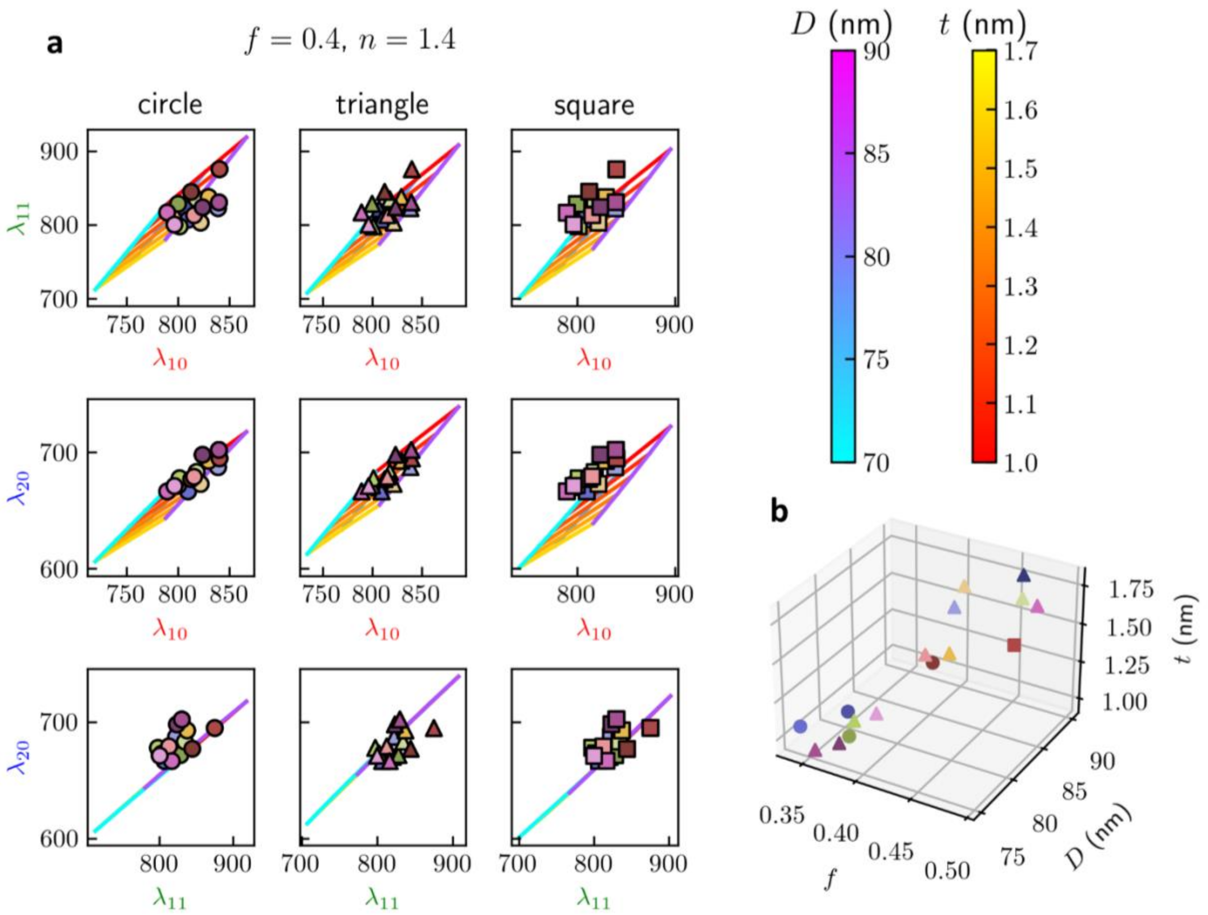

Fig. S6. a, Analogous plot to Fig. 5a, varying D instead of f. **b,** Freely optimized f, D, t for each NPoM with its likely shape (marker shape). The averages of D and t are taken from this plot for use in Fig. 5b and Fig. 5d respectively.

Fit of Au permittivity

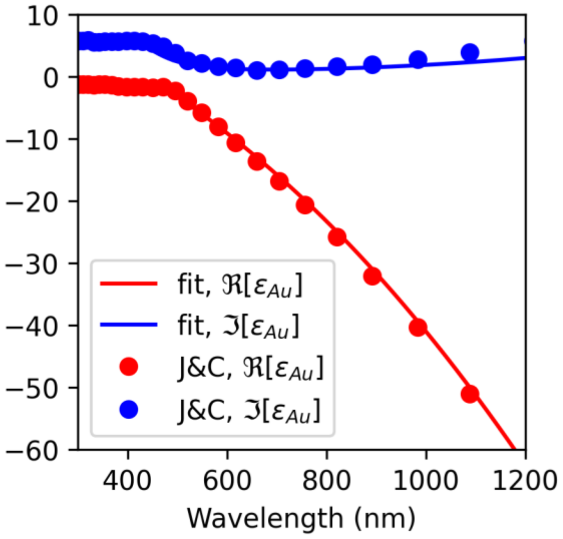

Fig. S7. Fitted real (red) and imaginary (blue) components of ϵ_{Au} to Johnson & Christy data [Ref 41, main text]. An excellent fit is obtained with a 2-pole Lorentz-Drude model.
